# Can sleep proﬁles predict autistic traits in siblings of children with autism spectrum disorder?

**DOI:** 10.5935/1984-0063.20200073

**Published:** 2021

**Authors:** Alshaimaa A. Naeem, Hala A. El-boraie, Tamer A. Abou-Elsaad, Mohammed E. Khater, Md. Dilshad Manzar, David Warren Spence, Seithikurippu R. Pandi-Perumal, Nevin Fayez Zaki

**Affiliations:** 1 Egyptian ministry of health, Egypt.; 2 Department of Psychiatry, Faculty of Medicine, Mansoura University, Egypt.; 3 Phoniatrics Unit, Department of E.N.T., Faculty of Medicine, Mansoura University, Egypt.; 4 Department of Nursing, College of Applied Medical Sciences, Majmaah University, Majmaah 11952, Saudi Arabia.; 5 Independent researcher, 652 Dufferin St., Toronto, Canada.; 6 Somnogen Canada Inc., College Street, Toronto, Canada.; 7 Sleep Research Unit, Department of Psychiatry, Faculty of Medicine, Mansoura University, Egypt.

**Keywords:** Sleep, Autistic Disorder, Autism Spectrum Disorder, Siblings of ASD, ASD, CAST, Autism

## Abstract

**Introduction:**

As the prevalence of ASD (autism spectrum disorder) continues to rise, so does the need to evaluate the impact of associated difficulties on both the diagnosed child and the immediate family.

**Objectives:**

The aim of the present study was to assess reports of sleep disturbance or abnormal sleep behaviours (sleep proﬁles) in the siblings of diagnosed autistic children (referred to throughout this study as high-risk siblings, or HR-sibs) and to determine if these sleep patterns correlated with evidence of disturbed sleep among their siblings who had full symptoms of autistic spectrum disorder.

**Material and Methods:**

This case control cross-sectional study investigated 64 autistic children, 80 HR-sibs, and 80 typically developing children. Each study subject was assessed for sleep problems and autistic traits through the use of a sleep-wake diary, a school sleep habit survey, and a childhood autism spectrum test.

**Results:**

Children with autism spectrum disorders and their HR-sibs showed no signiﬁcant differences regarding their sleep proﬁles. Typically, developing children had more middle insomnia than HR-sibs and had more wake latency.

**Conclusion:**

Increased risks for sleep problems in children with autism and their HR-sibs emphasized the importance of early screening for sleep problems in children with autism and their siblings.

## INTRODUCTION

Autism spectrum disorders (ASDs) are complex, pervasive, and multifactorial neurodevelopmental conditions. Observation of aberrant behavior forms the basis of diagnosis, with criteria focused on impairments in social communication and interaction, and restricted, repetitive patterns of behavior, interests, or activities^9^.

In the period when it was first identified, autism was considered a rare condition. Along with greater awareness of autism and its symptoms, this view has changed. The most recent prevalence rates in the United States estimate that 1 in 59 children have an ASD diagnosis^7^.

The risk of the development of ASD in siblings of children with ASD (hereinafter, high-risk siblings, HR-sibs) is estimated to be around 18.7%^19^. Besides, HR-sibs more frequently show subclinical features of ASD, also referred to as the broader autism phenotype (BAP)^19^. This includes delays in social communication such as the use of eye contact, gestures, and orientation to name^10^. Aside from BAP, HR-sibs without ASD also show more language difficulties, such as delays in receptive language^13^ or are delayed in their cognitive development during the first 3 years of life^4^. Thus, the developmental trajectories of HR-sibs are often characterized by early deficits, irrespective of a later ASD diagnosis. Commonly identified psychiatric and cognitive comorbidities with ASD include social anxiety disorder, oppositional defiant disorder, attention-deficit/hyperactivity disorder (ADHD), and intellectual disability^16^. Other frequently reported medical conditions include immune system abnormalities, gastrointestinal disorder, mitochondrial dysfunction, sleep disorders, and epilepsy^3,27^.

The prevalence of sleep problems among typically developing children is around 25% ^17^, while in the ASD population this proportion ranges from 50 to 80%^15^. Although sleep-onset issues and insomnia are often, the most common sleep problems reported by parents of children with ASD, night awakenings, poor sleep routines, and parasomnia are also frequently reported in this population^18^.

Among children with ASD, those who also have sleep problems score worse on socialization measures and exhibit significantly greater social skill deficits when compared to children with ASD who have no sleep difficulties. Greater communication impairments are demonstrated by children with ASD who exhibit increased sensitivity to environmental stimuli at bedtime and screaming during night awakenings. Fewer hours of sleep, screaming during awakenings, bedtime resistance, and settling difficulties have been correlated with higher rates of stereotypic behaviors and stricter adherence to non-functional routines^1,5^. Similar to children with autism, HR-sibs have been shown to have a higher risk of early insomnia, but to a lesser degree. The HR-sibs also suffer from more sleep problems, and particularly of parasomnias, than ASD children or typically developing children^8^. Increased rates of parasomnia may be explained by the following reasons: anxiety, sleep problems of their autistic sibling, and parents^14^ and potentially abnormal brain abnormalities as shown by electroencephalography.

Sleep problems in families with ASD children typically have broad effects in terms of parental distress and the functioning of the family. These may occur as a result of parents having to deal with the behavioral challenges that frequently occur among both the child and family who are experiencing sleep deprivation^21^.

There is a paucity of studies about the high-risk siblings of children with autism. This represents both an important clinical and research deficit inasmuch as the HR-sibs are at risk for emotional and behavioral problems^11^. These difficulties can have secondary effects since they are frequently associated with sleep problems, and thus it is reasonable to hypothesize that both ASD children, as well as their siblings who have few or no ASD symptoms, may both experience increased sleep problems. Given the limited research evidence in this area, and, further, because of the potential diagnostic value the presence of such symptomatology would possess for clinical management, the current study was undertaken to clarify these issues. The aims of the study, therefore, were to investigate the occurrence of sleep problems among children with autism, their siblings and typically developing children, and to determine if sleep problems were associated with autistic symptoms in siblings of children with autism.

## MATERIAL AND METHODS

### Participants

**Figure 1:**
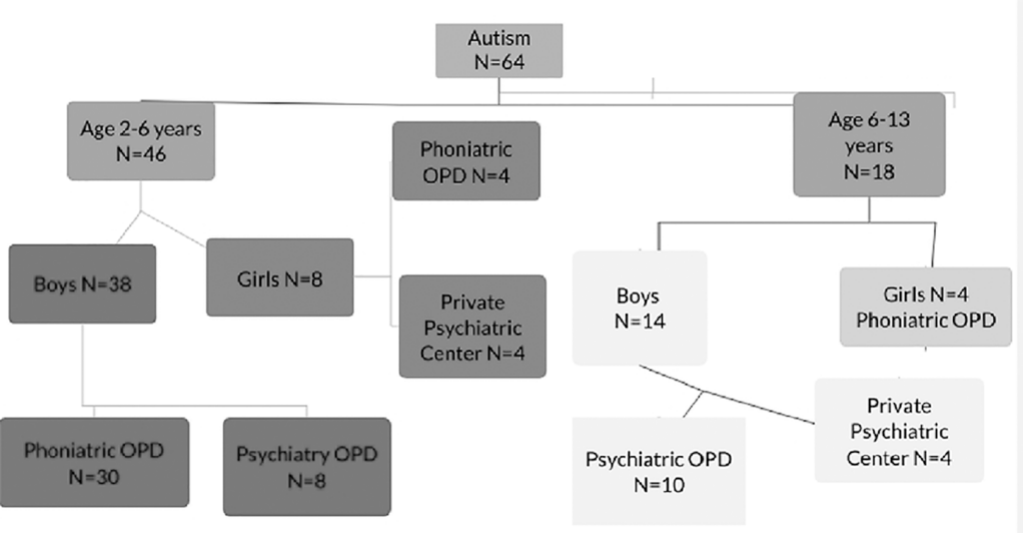
Sampling of the autism group.

**Figure 2:**
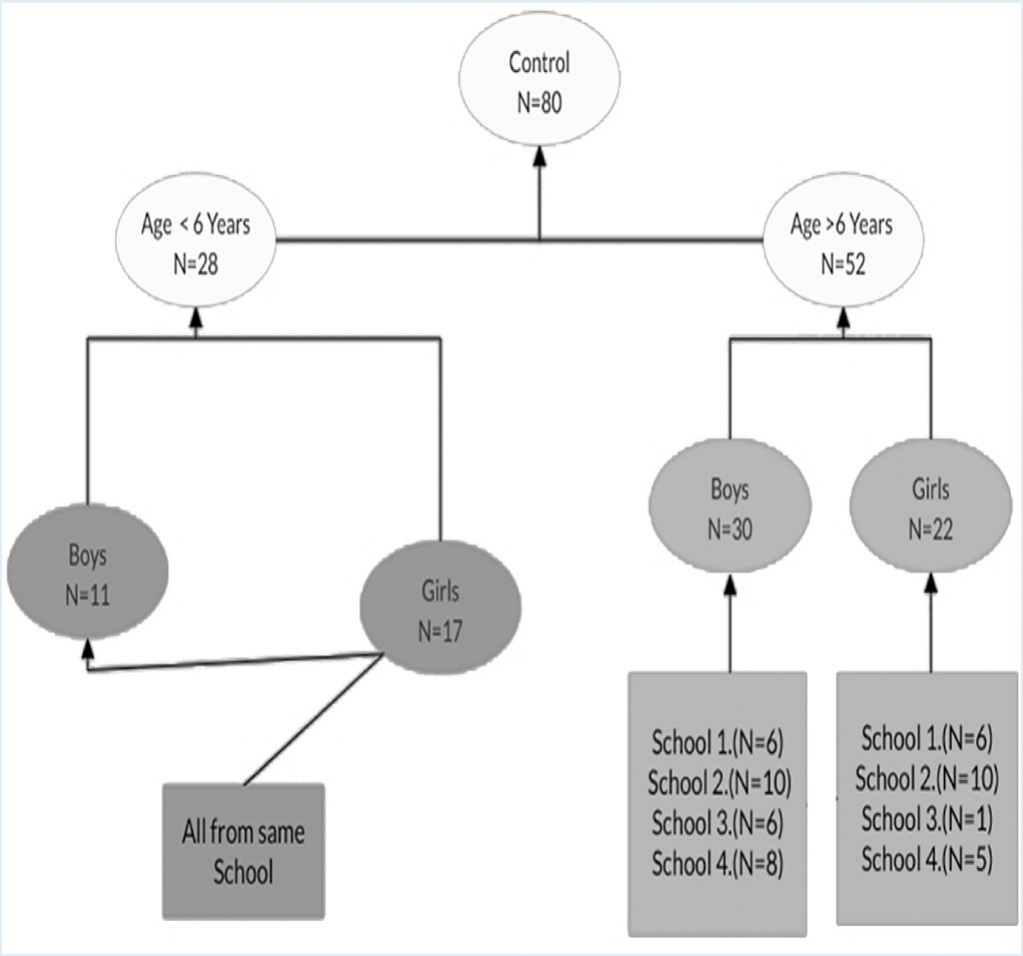
Sampling method for control group.

**Figure 3:**
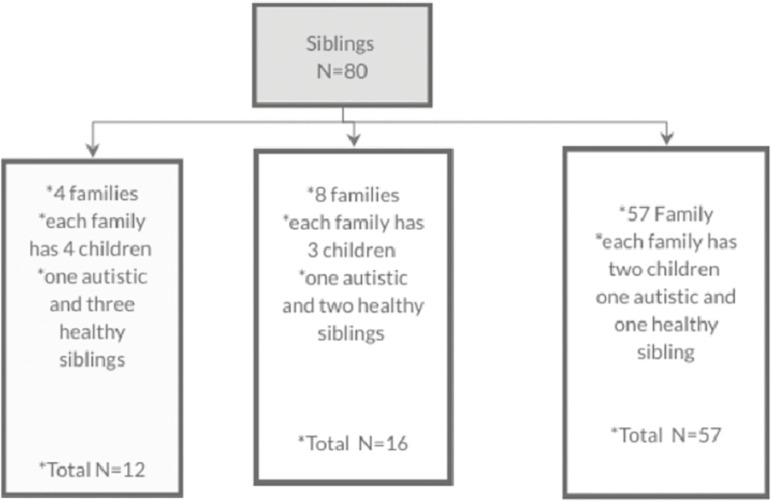
Sampling for the Autism sibling group

**Figure 4:**
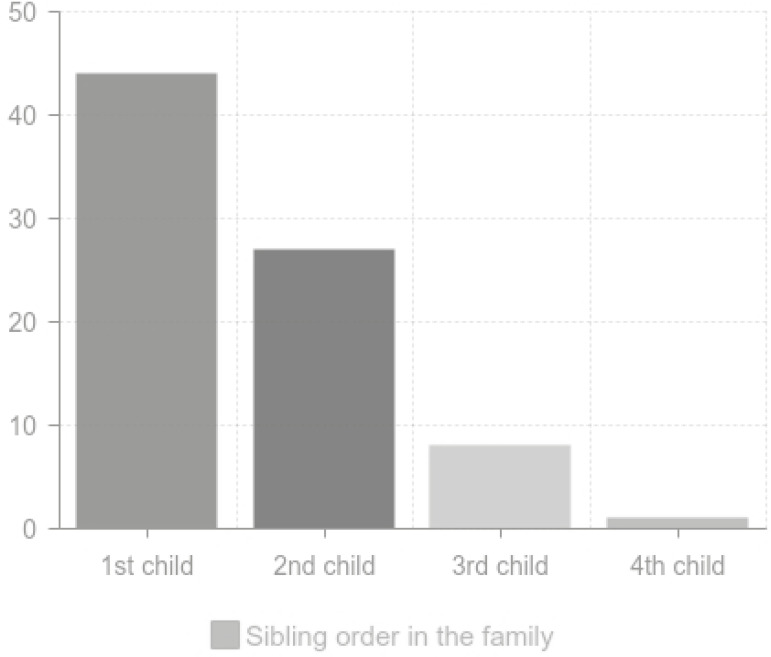
Caption birth order of the autism sibling group.

The study was carried out on children with autism spectrum disorder and on their siblings who had not been diagnosed as having autistic symptomatology. The third group of typically developing children (normal) were also studied. The subjects were classified into three groups: group I included, children with autism spectrum disorder (64 children); group II included siblings of children with autism spectrum disorder (80 children); and group III included normal children (80 children). Sampling distribution and recruitment steps of autistic can be summarized in Figure 1. Sampling of controls can be summarized in Figure 2, while the details of the sibling group can be seen in Figures 3 and 4.

The study sample was made up of 224 subjects; these consisted of 64 children with autistic spectrum disorder (age range 1.5-12 years). Senior psychiatrist already had diagnosed autistic children before being recruited in the study. Gender distribution of each group can be shown in Table 1A (group I), 80 children who were their non-symptomatic siblings (group II), and 80 typically developing (normal) children (group III). Children who were 6 years old or older enrolled as regular students in primary schools. All the study subjects were living with their biological families. If an autistic child had more than one sibling, all those who were appropriate for the study were included details of sibling recruitment can be seen in Figure X. The study also surveyed 80 normal children (males and females; age range 1.5-12 years) who were not suffering from any psychiatric, neurological, or medical disorders. All children in the normal group were aged 6 years or older and were enrolled as regular students in primary schools. All subjects were living with their biological parents/families.

**Table 1A.** Gender distribution in each of the study groups.

Variable	Group I (Autistic) N%	Group II (Siblings) N%	Group III (Control) N%
**Age group <6 years**			
Boys	38(82.6)	18(46.2)	11(39)
Girls	18(17.6)	21(53.8)	17(60)
**Total N**	46	39	28
**Age group >6 years**			
Boys	14(77.8)	27(65.9)	30(57.7)
Girls	14(22.2)	14(34.1)	22(42.3)
**Total N**	18	41	52

Inclusion criteria for siblings of children with autism spectrum disorder: both sexes, aged from 6 to 12 years, free from any comorbid medical illness, and both biological parents are present to avoid the effect of single-parent upbringing. Exclusion criteria for siblings of children with autism spectrum disorder: mental retardation, epilepsy, or any other neurological disease. Inclusion criteria for the normal control group: age and sex are matched with the ASD sample exclusion criteria for the normal control group: is receiving medical treatment, which might interfere with psychiatric assessment, any previous DSM IV-TR child psychiatric diagnosis, or positive family history of psychiatric disorders.

### MEASURES

All participants were subjected to the following:

1. The childhood autism spectrum test: the childhood autism spectrum test (CAST)^24^ (formerly the “childhood Asperger’s syndrome test”) is a 39-item, yes or no evaluation designed to be a parental self-completion questionnaire. The questionnaire was developed by ARC (the Autism Research Centre), in 2004, at the University of Cambridge, UK^24^, for assessing the severity of autism spectrum symptoms in children. The maximum score possible is 31, with a cut-off of 15 (indicating possible ASD or related social-communication difficulties). We downloaded the translated Arabic version from the Autism Research Center Website (https://www.autismresearchcentre.com/arc_tests) the questionnnaire was free to use from the developers.

It also contains a separate special needs section that asks about other comorbid disorders that the child might have.

The CAST measures difficulties and preferences in social and communication skills covering: initiation and maintenance of conversation and specific language difficulties; social interaction with adults and peers, including eye contact; choice of play activities; the presence of rigid or repetitive behaviors; choice of interests; and sharing interests with others.

CAST compared with other tools available showed success for identifying children at risk for AS and related conditions^24^. The test-retest reliability was tested in several research studies and proved to be good (Williams et al., 2006; Allison et al., 2007).

2. Sleep/wake diaries: sleep/wake diaries were provided to the parents or caregivers to record the children’s sleeping and waking times and other related information. The sleep diary comprised of the following components: the time the child woke up, whether he/she woke up spontaneously, by an alarm clock, or because of another (specified) disturbance; the time the child got out of bed, a brief description about how the child felt during the day (mood, drowsiness, etc., and, if possible, a rating of symptom severity on a scale from 1 to 5); the start and end times of any daytime naps and exercises; the name, dosage and time of any drugs used including medication, sleep aids, caffeine and alcohol; the time the child tried to fall asleep; the time the child thought sleep onset occurred, the presumed cause, number, time, and length of any night-time awakenings and activities during these moments, and the quality of sleep. The diary was used to screen sleep profile for those children younger than 6 years to whom other sleep screening questionnaires were not applicable.

3. School sleep habit survey: developed by Wolfson and Carskadon (1998)^26^, the School sleep habit survey is an eight-page, 63-item questionnaire designed to assess the sleep/wake habits and typical daytime functioning of high school students. The scale was translated into the Arabic language after approval from the original developer was obtained. Additionally, the adaptation of the questions to fit the usage of children from 6-12 years old was done during the translation process. Additionally, the research team helped the children in reading, understanding, and answering the survey several scales were created from items in the survey by the original survey inventors. These subscales were adopted by the research team in this study, they included: the depressed mood scale, sleepiness subscale, sleep/wake problems behavior scale, and the super science scale morningness/eveningness scale^6^. This scale is derived from responses to questions 47 through 56 of the school sleep habit questionnaire.

Response items for questions 47, 52, and 54 are coded from 5 to 1 with the first response item coded as 5 and the last response item coded as 1. Response items for questions 48, 53, and 55 are coded from 1 to 4 with the first response item coded as 1 and the last response item coded as 4. Response items for questions 49, 50, 51, and 56 are coded from 4 to 1 with the first response item coded as 4 and the last response item coded as 1. Item scores are summed to obtain a total scale score.

The section relating to sleepiness asks respondents to indicate whether or not they had struggled to remain awake in 10 different situations on a scale ranging from 1 (“no”) to 4 (“both struggled to stay awake and fallen asleep”). Total scores on this scale can range from 10 to 40, with higher scores indicating greater sleepiness. The scale was applied to children of 6 years and older similar to the study conducted by Russo et al., 2007^x^.

The sleep/wake problem behaviors scale queries the frequency of 10 different behaviors using a scale that ranges from 5 (“everyday”) to 1 (“never”), with possible total scores ranging from 10 to 50. Finally, the depressive mood scale consists of six items, with a response scale ranging from 1 (“not at all”) to 3 (“somewhat too much”).

Higher scores on this scale indicate more acute depressive symptoms. The school sleep habit survey was administered to participants who were attending primary schools (either from the control group or from HR-sibs siblings’ group).

### Data analysis

Data were analyzed using the IBM SPSS software package version 20.0. Qualitative data were described using the number and percent. Quantitative data were described using median (minimum, maximum, and interquartile range) for nonparametric data after testing for normality using the Kolmogorov-Smirnov test. A two-tailed-test of significance was applied, and the results were assessed at the 5% level of significance.

*Qualitative data:* chi-square test was used for comparison of 2 or more groups. Monte Carlo test was conducted as a correction for the chi-square test when more than 25% of cells have counted less than 5 in tables (>2*2). Fischer exact test was used as a correction for the chi-square test when more than 25% of cells have counted less than 5 in 2*2tables.

The chi-squared test for independence between two categorical variables uses a normal approximation to generate a *p*-value. The approximation is appropriate when the expected value under independence is large enough in each cell (say at least 5). For 2*2 tables, this assumption can be avoided by using Fisher’s exact test. However, for tables larger than 2*2, SPSS does not supply an exact test by default. Exact tests can be generated for ny size cross-tabulation by permutation, *SPSS provides permutation tests for all test statistics used for two-way classifications after clicking the ‘exact’ button in the crosstabs dialogue box and checking ‘exact’ or ‘Monte Carlo’ in the resulting sub-dialogue box.

*Quantitative data between two groups*:

1. Parametric variables: student t-test was used to compare 2 independent groups. One Way ANOVA test was used to compare more than 2 independent groups with post-hoc Tukey test to detect pair-wise comparison.
2. Non-parametric variables: Mann-Whitney U test was used to compare 2 independent groups. Kruskal Wallis test was used to compare more than 2 independent groups with the Mann Whitney U test to detect pair-wise comparison. Quantitative variables were undertaken to compare between more than two studied groups. A post-doc Tukey test for pairwise comparisons was then carried out. For testing of nonparametric quantitative variables, a Mann Whitney test was used, while a Kruskal Wallis test for nonparametric quantitative variables was used for comparisons of more than two studied groups. The Spearman correlation testing was used to correlate nonparametric continuous variables. Other tests including the chi-square test, the Fischer’s exact test, and the Monte Carlo test were used as appropriate to compare categorical variables.

### Procedure

The research plan was submitted for approval by the Medical Research Ethics Committee, Faculty of Medicine, Mansoura University, (Approval number MD/183). The study was explained to parents orally, and all of their questions were answered. Their written consent was obtained before assessment testing was carried out. The CAST (testing for possible ASD or related social-communication difficulties) and sleep-wake diary were completed by either of the parents.

The present study was carried out on siblings of children with an ASD who had been referred from the outpatient clinics “OPC” of Children Unit of Psychiatry Department and Phoniatrics unit of the ENT Department of the Mansoura University Hospital “MUH”. The study was also carried out on normal children who are studying at primary schools. These children served as control subjects for the study.

## RESULTS

Gender distribution of the recruited children can be seen in (Table 1A), it can be noticed that the majority of sample were boys. All of the autistic children had mild-severe level of ASD as determined by the CAST score in both age groups (Table 1B). About 5.1% of autistic siblings showed mild symptoms of ASD as determined by their CAST scores in both age groups (Table 1B). Understandably, there were no cases with even mild ASD symptoms among control children in both age groups (Table 1B).

**Table 1B.** Distribution of the three studied groups (autistic children, autistic siblings’ and control) with respect to their childhood autism spectrum test (CAST) interpretation.

Group		Group I Autistic children		Group IIAutistic siblings’		Group III Control	
		Frequency	Percentage	Frequency	Percentage	Frequency	Percentage
**<6 years**	Normal	0	0.0%	37	94.9%	28	0.0%
	Mild ASD	26	56.5%	2	5.1%	0	0.0%
	Moderate	17	37%	0	.0%	0	0.0%
	ASD						
	Severe ASD	3	6.5%	0	.0%	0	0.0%
**≥6 years old**	Normal	0	0.0%	39	95.1%	52	100%
	Mild ASD	6	33.3%	2	4.9%	0	0.0%
	Moderate	11	61.1%	0	0.0%	0	0.0%
	ASD						
	Severe ASD	1	5.6%	0	0.0%	0	0.0%

ASD: Autism spectrum disorder

### Sleep-wake diary: comparison between autistic children, HR-sibs and control group of fewer than 6 years

The comparisons revealed that no statistically significant differences existed between autistic children and their symptom-free siblings regarding sleep disturbances as measured by the sleep-wake diaries. Besides, no statistically significant differences in sleep behavior or experience were found between HR-sibs and control except for wake latency in the control group.

Correlation between the sleep-wake diary and CAST in autistic children and their siblings

In autistic children, a negative correlation was found between sleep time and CAST scores (Table 3). In HR-sibs, a positive correlation was found between wake after sleep onset scores and CAST scores. Moreover, a negative correlation was found between sleep time and CAST scores (Table 3).

**Table 3.** The correlation between the sleep-wake diary and CAST in autistic children and their siblings

Measures from	Childhood Autism Spectrum Test			
Sleep-wake diary				
	**<6 years**		**≥6 years**	
	**Group-I**	**Group-II**	**Group-I**	**Group-II**
	Autistic	Autistic	Autistic	Autistic
	children	siblings’	children	siblings’
Sleep time	-.332 *	-.327 *	-.053	-.203
Sleep onset	-.065	-.295	-.053	-.173
Sleep latency in minutes	.104	-.042	.478 *	-.182
Wake after sleep onset	-.016	.352 *	.025	-.183
Subjective sleep duration in hours	-.004	.010	-.129	-.188
				
Objective sleep duration	-.040	.083	-.260	-.158
Wake onset in hours	-.096	.132	.243	.126
Wake latency in minutes	.155	.209	.431	.000

* p<.05

### Study-2: sleep in autistic children, their siblings and control children of 6 years and above

#### Autistic traits in siblings of autistic children of 6 years and above

The comparison revealed that there was a statistically significant difference regarding CAST interpretation (*p*-value <0.05) between autistic children and HR-sibs (Table 1). Also, the comparison revealed that there was no statistically significant difference regarding CAST interpretation (*p-value* >0.05) between HR-sibs and the control group. Autistic children have mainly moderate ASD (Table 1).

#### Sleep-wake diary: comparison between autistic children, HR-sibs and control subjects aged 6 years and above

The comparison revealed that no statistically significant differences existed between autistic children and HR-sibs regarding disturbed sleep patterns, except for the greater sleep latency in the autistic children with a median range of 15 min in ASD children and 10 min in ASD siblings and a *p*-value of 0.04 (Table 4). Besides, the comparison revealed no statistically significant differences between HR-sibs and controls regarding the sleep-wake diary behaviors, except for wake and wake after sleep onset, which was comparatively increased in the control group with a *p*-value of (0.02-0.016, respectively) (Table 4).

**Table 4.** Comparison between autistic children, autistic siblings’ and control group of 6 years old and more regarding sleep-wake diary interpretation.

Group	Group I Autistic children	Group II Autistic siblings’	Z/t, p-value	Group II Autistic siblings’	Group III Control	Z/t, p-value
Sleep latency in min	15 (0-120) #	10 (0-45) #	z=2.03,	10 (0-45) #	10(0-30) #	z=0.51,
			p=0.04*			p=0.61
Wake after sleep onset in min	5 (0-90) #	0 (0-90) #	z=1.69,	0 (0-90) #	5(0-120) #	z=2.4,
			p=0.09			p=0.016*
Wake	1 (0-4) #	1 (0-3) #	z=1.30,	1 (0-3) #	1(0-4) #	z=2.33,
			p=0.19			p=0.02*
						
Subjective sleep duration in hours	9.01±1.3	8.5±1.3	t=1.35,	8.5±1.3	8.44±1.09	t=0.24,
			p=0.18			p=0.82
Mean±SD						
						
Objective sleep duration in hours	8.48±1.5	8.15±1.32	t=0.87,	8.15±1.32	8.07±1.1	t=0.28,
			p=0.39			p=0.78
Mean±SD						
Wake latency in min	0 (0-60) #	0 (0-60) #	z=0.27,	0 (0-60) #	0 (0-60) #	z=0.27,

# median (range)

### Correlation between the sleep-wake diary and CAST in autistic children and their siblings of 6 years and above

A positive correlation between sleep latency and the CAST was found (Table 3). There was no correlation between the sleep-wake diary and CAST in the HR-sibs group (Table 3).

### Depressive mood scale, sleepiness scale, sleep-wake problems behavior scale, super- science mornings evening scale: comparison between HR-sibs and control group of 6 years old and more

The comparison revealed no statistically significant differences among the studied groups in terms of depressive mood. Both the autistic children’s sibs and the control group subjects fell mainly within the mild depression group (72.5% and 69.2%, respectively) (Table 5). The comparison revealed that there was no statistically significant difference regarding the school sleep habit survey (sleepiness scale interpretation) between ASD sibs and the control group. The two groups showed that most of the children had no sleepiness during the daytime, although a small percentage of children did have mild sleepiness during the daytime (ASD sibs 17.5%, control 21.2%) (Table 5).

**Table 5.** Depressive mood scale, sleepiness scale, sleep-wake problems behavior scale, super-science mornings evening scale. Comparison between autistic siblings’ and control group of 6 years old and more.

Group	Group II Autistic siblings’		Group III Control		Χ2	p-value
Depressive mood scale	Frequency	Percentage	Frequency	Percentage		
Normal	9	22.5%	11	21.2%	.69	.71
Mild depression	29	72.5%	36	69.2%		
Severe depression						
Sleepiness scale	2	5.0%	5	9.6%		
No sleepiness	32	80.0%	40	76.9%	.22	.89
Mild sleepiness	7	17.5%	11	21.2%		
Greater sleepiness						
Sleep-wake problems behavior scale	1	2.5%	1	1.9%		
No problems	29	2.5%	45	6.5%	2.83	.09
Mild problems	11	7.5%	7	3.5%		
Moderate problems	0	0.0%	0	0.0%		
Severe problems	0	0.0%	0	0.0%		
Super-science mornings evening scale						
Maximum evening preference	0	0.0%	0	.0%	.53	.47
Both preference	29	72.5%	34	5.4%		
Maximum morning preference	11	27.5%	18	4.6%		

The comparison revealed no statistically significant differences among studied groups regarding the school sleep habit survey (sleep-wake problems behavior scale interpretation). The percentage of the control group and HR-sibs who had no problems related to sleep-wake problems were (6.5% and 2.5%, respectively), although a few children did show evidence of mild difficulties in this regard with the autistic sibs showing a higher percentage than the controls (7.5% and 3.5%, respectively) (Table 5). The comparison revealed no statistically significant differences between HR-sibs and controls regarding preferences for owl-lark schedules, as shown by the super-science mornings/evenings scale. Children in both groups prefer to work during both (evenings and mornings) to the same degree, as some children prefer working in the morning more (Table 5).

### Correlation between the School sleep habit survey and CAST in autistic children and their siblings of 6 years and above

In HR-sibs, a positive correlation was found between depressed mood scale scores and CAST normal scores. Also, a positive correlation was found between the super-science mornings/evenings scale (owl-lark) scores and CAST normal scores (Table 6).

**Table 6.** The correlation between the school sleep habit survey and CAST regarding autistic siblings' and control group of 6 years old and more.

Measures from school sleep habit survey	CAST	
	Group II autistic siblings	Group III controls
Depressed mood scale	0.394	0.182
Sleepiness scale	0.216	0.104
Sleep-wake problems behavior scale	0.249	0.057
Super-science morningness-eveningness scale (owl-lark)	0.441	-0.058

## DISCUSSION

Although several studies in western countries have investigated sleep problems among children with ASD, the current study is one of the few studies on sleep problems of Egyptian children with ASD. Most importantly, to our knowledge, this is the first study in Egypt to investigate sleep profiles in HR-sibs and their relation to autistic traits. Studies comparing autistic individual with their normal siblings produce conflicting results, some studies found that autistic children show worse symptoms, and some other studies showed no differences between the autistic subjects and their typically developing siblings. In a study by Shivers et al. (2019)^25^, ASD-siblings showed worse internalizing behavior, psychological functioning, beliefs, social functioning, beliefs about disability, anxiety symptoms and depression symptoms but the sleep profile of both groups was not studied.

The principal finding of our study was that no significant differences were shown to exist between children with autism spectrum disorders and their HR-sibs, nor between these two groups and control subjects in terms of their sleep profiles. More specifically, no differences were found between autistic children and their siblings concerning the variables of sleep duration, wake latency, wake after sleep onset, and nor wake.

These findings may be explained partially by their neurobiological similarity, or by behavioral changes and aggressive behavior of autistic children, which may make it difficult for their siblings to have a good night’s sleep. These findings were further elaborated in a research by Ruzich et al. (2016)^22^, which found that siblings as a group have intermediate levels of autistic traits compared to control individuals and participants with autism the low-scoring sibling group is more similar to typical controls while the high-scoring group is more similar to the autism clinical group.

Similar to autistic children, the HR-sibs had a higher risk of early insomnia, but to a lesser degree than autistic children did, and this may be explained by the daily fixed routine of autistic children and their poor ability to understand different environmental cues. Besides, in our research, we did not find a significant difference between the HR-sibs and the control group regarding sleep latency nor in sleep duration. Nevertheless, we found that typically developing children (normal control group) had more middle insomnia than the HR-sibs, and had more wake latency. These findings may be explained partially by a lack of adherence to sleep schedule and an inconsistent parenting style.

Our findings were consistent with previous reports such as those of Gau et al. (2012)^11^, in as much as they did not find significant differences in sleep duration between children with autism and typically developing children. Children with autism and their HR-sibs had more fixed sleep schedules and more sleep problems, compared to the healthy children in non-autistic families. Children with autism tended to have early insomnia, middle insomnia, sleep-wake schedule disorders, and daytime napping than the controls^2^. Similarly, Chou et al. (2012)^8^ showed that children with autism had more sleep problems, including insomnia, sleep-wake schedule disorders, and daytime napping.

Their unaffected siblings also had more risk of insomnia, sleep talking, and nightmares, compared to the typically developing children. Their findings of increased risks for sleep problems in both children with autism and their unaffected sibling suggest that parenting counseling should be included in intervention of sleep problems in children with autism and their siblings.

In our research, HR-sibs and typically developing children showed more mild depression, no sleepiness during daytime, mild sleep-wake problem behavior, and higher morningness- eveningness preference than a primarily morning preference. These findings may be explained by more parental care and support for autistic children than their siblings and behavioral changes in autistic children.

These findings were consistent with the study showing that parental monitoring has been reported to have associations with morningness-eveningness, bedtime, and sleep duration on weekdays. Less authoritarian parenting in the sibling group, as well as the erratic sleep/wake patterns of their autistic siblings and parents, may contribute to later rise time on weekdays in HR-sibs^2^.

## CORRELATION BETWEEN SLEEP PROBLEMS AND AUTISTIC TRAITS

In autistic children, sleep time is related to fewer symptoms of autism, while early insomnia tends to increase with an increase in autistic symptoms. In HR-sibs, sleep time is related to fewer autistic traits, while middle insomnia increases with an increase in autistic traits. In addition, in siblings, depressed mood, and morningness-eveningness preference is related to more autistic traits.

This may be explained by findings that, with increased autistic symptom severity, affected children showed more repetitive and ritualistic behaviors, cognitive inflexibility, stereotypies, and insistence on sameness. Being forced to defect from these routines has been found to cause significant distress, and can delay sleep time or cause insomnia.

In our findings, sleep profiles in HR-sibs did predict autistic traits. However, these findings should be considered exploratory because sleep problems and autistic symptoms scales were based on parental reports only, and were without confirmation by polysomnography or actigraphy. Furthermore it was found that in families where the child diagnosed with ASD was older than the normal sibling, the impact of the diagnosed child was stronger autistic older brother’s behaviors were very likely to be imitated by normal younger siblings, thus no difference was found between both groups^20^. Similarly sleep and behavior problems in the autism siblings were not associated with subclinical autism features or developmental functioning, while parent reports of social and communication concerns robustly associated with sleep and behavior problems, those rated by expert examiners were not^23^.

### Study limitations

There are several methodological strengths in this current study. First, this study compared sleep problems among children with autism, their HR-sibs and typically developing children in one study, which provides novel information about the sleep problems in the unaffected sibling of children with autism.

Second, we tried to explore the relation between sleep profile and autistic traits in HR-sibs. Considering the scarce research investigating siblings of autism children.

However, it has significant limitations; first, all used scales in this study were completed by parents, which may have been the source of recall bias, inasmuch as parents are usually more alert to the condition of the sick child than among the rest of the siblings.

Furthermore, the level of parental stress was not measured since more stressed parents tend to report more problems when asked about their children. To increase the validity of the assessment, the use of other informants, such as a teacher, might have been useful, and the longitudinal design of the study can be more generalizable. Second, both the sleep profile and reported problems were assessed by subjective measures only, rather than by objective measures. Additionally inclusion of more than one HR-sib for each autistic child brings a bias as sleep disorders also have genetic basis but the direction of causality could not be determined by a cross-sectional study. Third, lack of information existed concerning the presence of other psychopathologies, a potential confound inasmuch as children with autism tend to have emotional and behavioral problems, which could have impacted their sleep quality. A fourth limitation was the study’s small sample size, which could have limited the validity of the between-group differences.

## CONCLUSION

Autism spectrum disorder has become prevalent more than expected. It does not only affect the child but also various impacts are seen on the family members, including parents and siblings. The need to evaluate these associated difficulties on both the diagnosed child and the immediate family is essential. This study assumed that there would be a common pattern of sleep difficulties manifesting in both the autistic candidate and his healthy siblings.

The present study aimed to assess reports of sleep disturbance or abnormal sleep behaviors (sleep profiles) in the siblings of diagnosed autistic children (referred to throughout this study as high-risk siblings, or HR-sibs), and to determine if these sleep patterns correlated with evidence of disturbed sleep among their siblings who had full symptoms of autistic spectrum disorder. To fulfill the aim a case-control cross-sectional study was designed. We investigated 64 autistic children, 80 HR-sibs, and 80 typically developing children. Each study subject was assessed for sleep problems using a sleep diary and school sleep habit questionnaire. Autistic traits were examined using the child autism spectrum test. Results revealed that children with autism spectrum disorders and their HR-sibs showed no significant differences regarding their sleep profiles. Typically, developing children had more middle insomnia than HR-sibs and had more wake latency.

The current study sheds the light on the importance of screening for sleep problems in children with autism and their healthy siblings and incorporates this step into the routine practice and interviewing of ASD subjects and their families.

## Figures and Tables

**Table 2.** Comparison between autistic children, autistic siblings’ and control group of less than 6 years old regarding sleep-wake diary interpretation:

Group	Group I Autistic children	Group II Autistic siblings’	Z/t, P value	Group II Autistic siblings’	Group III Control	Z/t, P value
Sleep latency in min	15.0 (0-80) #	15.0 (0-30) #	z=1.55,	15.0 (0-30) #	15.0 (0-30) #	z=0.28,
			p=0.12			p=0.78
Wake after sleep onset in min	7.5 (0-90) #	10 (0-120) #	z=1.15,	10 (0-120) #	10 (0-120) #	z=0.35,
			p=0.25			p=0.73
Wake	1(0-10) #	2 (0-5) #	z=0.93,	2 (0-5) #	1 (0-4) #	z=1.31,
	9.44±1.4	8.53±1.6	p=0.35	8.53±1.6		p=0.19
Subjective sleep duration in hours			t=0.72,		9.2±1.5	t=1.79,
			p=0.47			p=0.07
Mean±SD						
	8.86±1.3	8.09±1.6		8.09±1.6		t=1.78,
Objective sleep duration in hours			t=0.34,		8.76±1.4	
			p=0.74			p=0.08
Mean±SD						
						
Wake latency in min	0(0-30) #	0 (0-30) #	z=1.17,	0(0-30) #	10 (0-60) #	z=2.42,
			p=0.24			p=0.02*

# Median (range)
